# Role of offending out-door aero-allergen and CD14 C(-159)T polymorphism in development and severity of asthma in a Kolkata patient population

**DOI:** 10.4314/ahs.v17i4.18

**Published:** 2017-12

**Authors:** Shampa Dutta, Priti Mondal, Nimai Chandra Saha, Saibal Moitra, Sanjoy Podder, Amlan Ghosh, Goutam Kumar Saha

**Affiliations:** 1 Post Graduate Department of Zoology, Barasat Govt. College; 2 Department of Vice-Chancellor's Secretariat, The University of Burdwan; 3 Allergy and Asthma Research Centre, Kolkata; 4 Department of Life Sciences, Presidency University; 5 Department of Zoology, Calcutta University

**Keywords:** Asthma, aero-allergen, skin prick test, total IgE, CD14 gene polymorphism

## Abstract

**Objective:**

Present study involved identification of offending out-door aero-allergens and associated genetic pathway in nasso-bronchial asthma among Kolkata population.

**Methods:**

Skin-prick test was done among 950 asthmatic patients against 11 common aero-allergens and total serum IgE concentration was measured. PCR-RFLP was done in patients and non-asthmatic control (n=220 in each) to characterize functional polymorphism, C(-159)T, of CD14, a positional candidate gene for allergy. Association of genetic polymorphisms was made with clinico-pathological conditions.

**Results:**

We identified *Cocos nucifera* as the most common aero-allergen sensitizer among atopic patients in Kolkata. Patients with childhood-onset asthma were significantly more sensitive towards aero-allergens and had significantly higher serum IgE level than those of adult-onset (p< 0.0001). No significant difference was found in distribution of SNP genotypes of CD14 among case and control (p=0.178). However among patients, frequency of C allele is significantly higher in childhood-onset group than that of adult-onset and concordantly in former CC genotype was associated with significant higher level of serum IgE than CT and TT.

**Conclusion:**

In Kolkata, pollen is a common out-door aero-allergen and *Cocos nucifera* is predominant among pollens. Childhood-onset and adult-onset of asthma showed significant difference in allergen sensitivity as well as genetic background with respect to CD14 polymorphism.

## Introduction

Asthma is the most common type of atopic manifestation characterized by respiratory symptoms, narrowing of airways, and inflammation^1^. Prevalence of asthma is gradually increasing worldwide including developing countries and in India alone, roughly 15% of the people suffer from this disease^2^,^3^. Development of asthma in individual depends on both environmental stimuli like aero-allergen and genetic factors^4^. Exposure of aero-allergen serves as a trigger for worsening of asthma and therefore identification of disease specific allergen is important for designing avoidance strategy^5^. Airborne pollens and molds produced by flowering plants and fungi are the most important factors of asthma and India being a climatically diverse country supports huge diversity of such aero-allergens in different regions. In asthma, atopic response is characterized by allergic inflammation induced by Th2 (T-helper type-2) derived cytokines and production of allergen specific immunoglobulin-E (IgE)^6^. The gene encoding Cluster Differentiation antigen (CD14) is localized on chromosomal 5q31.1 region which is associated with both asthma and total serum IgE concentration^7^. Actually CD14 is a pattern recognition factor that plays a central role in innate immunity through recognition of bacterial lipopolysaccharide (LPS) leading to Th1 differentiation and suppression of Th2-dependent IgE responses^8^.

CD14 protein is found either as membrane molecule (mCD14) expressed primarily on the surface of monocytes /macrophagesor as soluble form (sCD14) in serum^9^. Regulation of CD14 gene expression and serum level of sCD14 might affect the proportion of Th1 to Th2 cells, thereby influencing IgE responses and associated inflammatory phenotype in asthma. Thus, alterations of CD14 expression appear to be important in asthma, and is likely to be regulated, at least partially, at the gene level. A genetic variant in the promoter region of the CD14, C(-159)T or rs2569190 has been found to be associated with altered levels of sCD14 and total serum IgE^9^. Role of CD14 polymorphism in asthma is widely explored in different ethnic population with diverse success i.e. Baldini et al (1999)^9^ reported association of CC homozygote with higher serum IgE level compared to CT and TT genotypes in atopic non-Hispanic white children. Similar findings were also reported by Buckova et al (2003)^4^ in Czech, Koppelman et al (2001)^10^ in Dutch and Leung et al (2003)^11^ in Hong Kong Chinese subjects.

On the contrary, Kedda et al (2005)^12^ found no association of the polymorphism with asthma in Australian subjects and Martinez et al (2007)^13^ proposed association of both C and T alleles of the polymorphism with allergic manifestation depending upon level of allergen exposure. There is paucity of data regarding aero-allergen sensitization among atopic patients in the Kolkata metropolitan area, particularly in asthma and CD14 polymorphisms in the same population. This study was undertaken to identify the common aero-allergen sensitizers among atopic patients and explore association of CD14 polymorphisms with disease onset and severity in the Bengali population of Kolkata.

## Methods

### Study subjects

**i. Selection of patients' and control subjects:** The study group composed of 950 patients clinically diagnosed as suffering from bronchial asthma at the Outpatient Department of the Allergy & Asthma Research Centre, Kolkata from 2010–2013.

**Inclusion criteria:** Individuals diagnosed as asthmatic patients on the basis of the criteria mentioned earlier^14^ were case subjects. Briefly individuals having at least 6 positive among the 8 symptoms (presence of wheezy dysponea; presence of frequent cough; presence of ronchi; showing intervals of relative/ complete freedom from symptoms; reports of onset of asthmatic bouts at an early age; with personal history of atopic diseases such as infantile eczema, hay fever and or urticaria; having family history of atopy; report of definite history of allergy to inhalant allergen, particularly to house dust) were considered as suffering from bronchial asthma that later confirmed by Spirometry. Individuals accompanying the case subjects, but not blood relative to the later, were selected as control. In total 220 healthy unrelated individuals (120 male and 100 female) belonging to the same age group and locality (Kolkata) of case and without any history of atopy were selected as control subject. The detailed information of case and control individuals is described in table-1a and b.

**Table-1a:** Detailed information of the case and control subjects

Characteristics		Case	Control
**Sex**	Male	550 (58%)	125 (57%)
	Female	400 (42%)	95 (43%)

**Age**	12 (Child)	158 (17%)	0 (0%)
	13–19 (adolescent)	60 (6%)	31 (14%)
	20 (Adult)	732 (77%)	189 (86%)

**Bronchial asthma (Doctors diagnosis)**		950 (100%)	0 (0%)

**Age of Onset**	Child hood onset (<20 years)	432 (45%)	0 (0%)
	Adult Onset (≥20 years)	518 (55%)	0 (0%)

**Disease severity**	Mild (Persistent)	219 (23%)	0 (0%)
	Moderate (Persistent)	295 (31%)	0 (0%)
	Severe (Persistent)	436 (46%)	0 (0%)

**Other atopic** **symptoms**	Allergic rhinitis or Urticaria	493 (52%)	0 (0%)
	Both	94 (9.8%)	0 (0%)
	None	363 (38.2%)	220 (100%)

**Table-1b:** Criteria for diagnosis of asthma severity

	Persistent Asthma		
	
	Mild	Moderate	Severe
Asthma Symptoms	≥3days/week	Daily	Throughout the day
Night time awakenings due to asthma	3–4 nights/month	>1 night/week	7 nights/week
Frequency of Short- acting beta2 agonist use	≥3days/week	Daily	Several times a day
Interference with normal activity	Minor limitation	Some limitation	Extreme limitation
% predicted FEV1 (mean±SEM)	80.3±1.5	72.5±1.9	58.1±2.8

**Exclusion criteria:** Cases with other organic and systematic diseases such as hypertension, diabetes, and also pregnant and lactating females were excluded. The personal and/or family history of each individual patient was recorded in a well prepared questionnaire and a complete record of the patients' physical and clinical condition was also maintained. Stool examination was done for three consecutive days against each case subjects to exclude possible interference of parasitic infections in the total IgE level of sera.

ii. **Patient population:** 950 asthmatic patients were enrolled into the study group of which 550 were male and 400 female. Each category was again stratified according to age groups i.e. child (≤12Years), adolescent (12–19 years) and adult (≥20 years), according to age of asthma onset i.e. childhood onset (< 20 years) and adult onset (≥20 years) and also according to disease severity i.e. mild, moderate and severe (Table-1a and b).

All (950) have been diagnosed to suffer from allergic asthma of which 61.8% of patients had also concomitant allergic rhinitis or urticaria or both.

### Allergy skin tests

Skin prick test of 950 patients against 6 types of pollen and 5 types of molds (obtained from Credisol India Ltd. Mumbai) were done as described earlier^14^. The allergens were selected on the basis of their predominance in environment of Kolkata metropolis. The interpretation of skin prick test result was done following the method of Grater et al (1982)^15^. Only 2+ and above reactions (2+ to 3+) were considered clinically significant.

### Quantification of total IgE level

Estimation of total serum IgE was carried out in 950 patients by Enzyme Immuno Assay (EIA) using chemicals Pathozyme Immunoglobulin (Ref: OD 417) supplied by Glaxo Smithkline Pharmaceuticals Ltd., Mumbai. The concentration of IgE is directly proportional to the color intensity of the test and the assay was calibrated against the WHO^16^ standard for IgE.

### Genotyping of rs2569190

This was done in 220 asthmatic individuals (case) all of which were skin test positive and in an equal number of healthy unrelated control.

**a. Genomic DNA isolation:** Genomic DNA was isolated from peripheral blood leukocytes by the proteinase-K digestion of cells followed by phenol/chloroform extraction according to a standard method^17^.

**b. PCR-RFLP:** Polymerase chain reaction (PCR) was carried out in a volume of 20 µl reaction mixture containing 50 ng genomic DNA, 4 pmol of each primers (Forward, 5′-GTGCCAACAGATGAGGTTCAC-3′ and reverse 5′-CCTCTGTGAACCCTGATCAC-3′), 0.2 mM of each deoxynucleotide triphosphates (Sibenzyme, Russia) and 1 unit of Taq polymerase (Sibenzyme, Russia). Following PCR, the product was digested with 2 units of AVAII endonuclease (New England Biolabs) for overnight, electrophoresed on 2% agarose gel and visualized by ethidium bromide staining on a gel documentation system (Bio-Rad GS-800). Ava II is specific for the sequence GGTCC which is present in PCR products containing only CD14 (-159) T alleles. The PCR product is 497 base pairs in length, digestion of which yielded bands of 497 base pairs in CC homozygotes, 144 and 353 base pairsin TT homozygotes, and all the three bands (144, 353 and 497 base pairs) in heterozygotes.

**c. DNA sequencing:** Representative samples for each genotype from case and control were subjected to auto-sequencing for validation. Both strands of each representative genotypes were subjected to sequencing using ABI PRISMTM BD Terminator Cycle Sequencing kit (PE Applied Biosystems, Foster City, CA, USA) and electrophoresed in 3100-Avant Genetic Analyzer (PE Applied Biosystems Inc, USA). The obtained electropherograms were analyzed using the sequencing analysis software (version 1.01, Applied Biosystem). Identification of specific sequence variations in the CD14 promoter region (-159 C/T) was done by aligning the forward and reverse complement sequences with the corresponding reference sequences (ensemble.org) using the Clustal W multiple alignment software program (European Bio Informatics Institute).

### Data analysis

Chi-square test, student t-test and Analysis of variance (ANOVA) were used to compare the allergic responses towards different aero-allergens, total serum IgE level and differences in genotype or allele frequencies of CD14 polymorphism in different age groups, genders etc. ANOVA was used to compare serum concentration of total IgE among CD14 genotypes in different groups with specific allergic phenotypes. Odds ratio (OR) and confidence intervals were calculated. P-values less than 0.05 were considered to be significant. All the statistical analysis was performed using statistical program Graph-Pad PRISM (version-5, 2007). For case control study of CD14 polymorphism, the power was estimated using Genetic power calculator^17^. Power was computed for an OR (Odds ratio) of approximately 0.8 for the SNP and it was 34%. Allele frequencies were estimated as the following formula: (frequency of homozygotes) + 1/2 (frequency of heterozygotes).

### Ethical approval

The Human Ethics Committee of Allergy and Asthma Research center, Kolkata approved this study with reference number CREC-AARC Ref: 03/200012. Informed written consent was obtained from all the participating individuals.

## Results

### Skin prick test

Of 950 patients, 638 (67%) gave positive immediate prick test reactions to at least one of the 11 aero-allergen sensitizers; in the remaining 312 (33%), there was no immediate skin reactivity (Table-2).

**Table-2:** Result of skin prick test for pollen and molds.

Skin prick test (positive)				
		Male	Female	p value
Total	638/950 (67%)	377/550 (68%)	261/400 (65%)	0.285
Pollen	*Cocos nucifera* (300/638, 47%)	190/377 (50%)	110/261 (42%)	0.04*
	*Caesalpinia sp* (285/638, 45%)	164/377 (44%)	121/261 (46%)	0.47
	*Carica papaya* (253/638, 40%)	148/377 (39%)	105/261 (40%)	0.8
	*Azadirachta indica* (241/638, 38%)	142/377 (38%)	99/261 (38%)	0.945
	*Cynodon dactylon* (231/638, 36%)	150/377 (38%)	81/261 (31%)	0.023*
	*Peltophorium sp* (207/638, 32%)	122/377 (32%)	85/261 (32%)	0.956
Mold	*Aspergillus fumigatus* (115/638, 18%)	78/377 (21%)	37/261 (14%)	0.035*
	*Alternaria alternata* (110/638, 17%)	67/377 (18%)	43/261 (16%)	0.669
	*Aspergillus niger* (99/638, 16%)	68/377 (18%)	31/261 (12%)	0.034*
	*Penicilium sp* (91/638, 14%)	63/377 (17%)	28/261 (11%)	0.033*
	*Aspergillus tamari* (76/638, 12%)	47/377 (12%)	29/261 (11%)	0.603

* P < 0.05

The most common aero-allergen sensitizers were pollen, of which most predominant one was *Cocos nucifera* (47%) followed by *Caesalpinia sp* (45%), *Caricapapaya* (40%), *Azadirachta indica* (38%), *Cynodon dactylon* (36%) and *Peltophorium sp* (32%). Amongst mold category, most predominant one was *Aspergillus fumigatus* (18%) followed by *Alternaria alternata* (17%), *Aspergillus niger* (16%), *Penicilium sp* (14%) and *Aspergillus tamarii* (12%). While considering the aero-allergen sensitizers with respect to severity of reactions, *Cocos nucifera* was found to be most potent i.e. 7% (66/950) of patients showed 3+ or more sensitivity to it, followed by *Caesalpinia* (5%, 47/950) and *Peltophorium* (3%, 29/950) (Data not shown). No significant difference in the distribution of overall skin test positive individuals was found between male and female (p=0.285). However, when allergen sensitizers were considered individually, frequency of sensitive individuals was found to be significantly more among males than females for *Cocos nucifera*, (p=0.04) and *Cynodon dactylon* (p=0.023) among pollen and *Aspergillus fumigatus* (p=0.035), *Aspergillus niger* (p=0.034), and *Penicilium* (p=0.033) among molds (Table-2).

**Figure 1:**
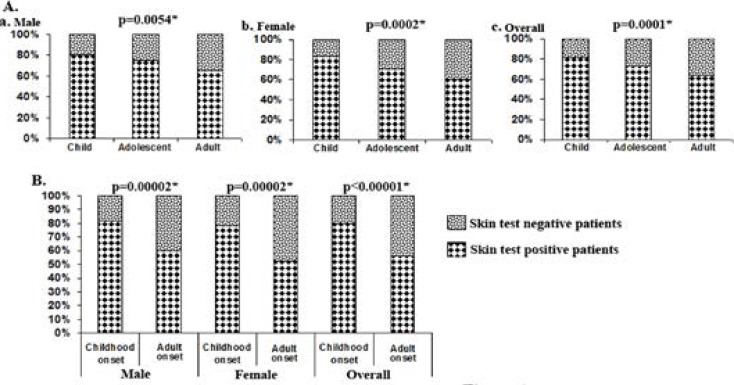
Relative proportion of skin test positive to skin test negative patients in child (≤12years), adolescent (13–19 years) and adult (≥20 years) (A) and according to age at onset of bronchial asthma i.e. childhood onset (<20 years) and adult onset (≥20 Years) (B). * P<0.05 (Chi-square test).

The frequency of skin test positive individuals was highest among children, which decreased gradually and significantly in adolescents and adults (Figure-1a). Furthermore, childhood onset group had significantly higher frequency of skin test positive individuals than that of adult onset (Figure-1b).

### Total IgE level

**Figure-2:**
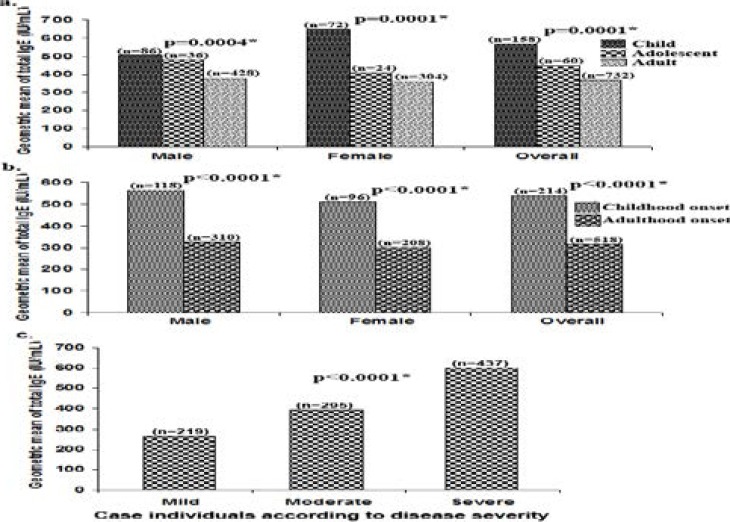
Geometric mean of total IgE level in child (≤12years), adolescent (13–19 years) and adult (≥20 years) (a) according to age at onset of bronchial asthma i.e. childhood onset (<20 years) and adult onset (≥20 Years) (b) and according to disease severity (c). * P<0.05 (analysis of variance and t-test).

The average IgE level of total 950 patients was 528 IU/mL (95% confidence interval 505–551 IU/mL). No significant difference in the mean IgE level was found among male and female concordant with the result of skin prick test. For both sexes, a significant negative correlation of total IgE level was found with the age of patients i.e. Children have the highest level of total IgE followed by adolescents and adults (Figure-2a). We also observed significantly higher IgE levels in patients with childhood-onset of asthma compared to those of adult-onset (Figure-2b) and across the patients with increase of severity of the disease (Figure 2c)

### Genotype frequency of the CD14 promoter region

**Figure-3:**
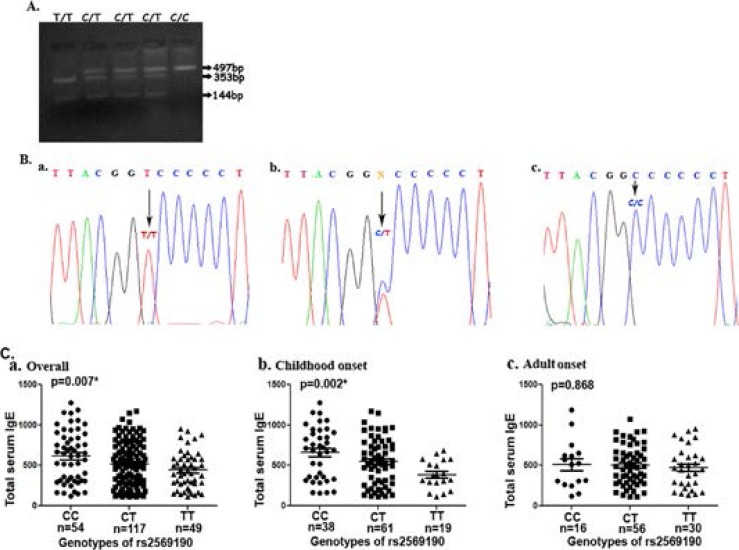
Genotyping of rs2569190 and its association with total serum IgE level. A. Detection of rs2569190 polymorphism by restriction fragment length polymorphism (RFLP) analysis. CC homozygote is in lane 5; TT homozygote is in lane 1 and CT heterozygotes are in lanes 2, 3, and 4. B. Representative chromatogram showing rs2569190 polymorphism and arrows indicate the polymorphic locus; CC homozygote (a), CT heterozygote (b) and TT homozygote (c). C. Scatter diagram showing total IgE levels in IU/ml and association with three rs2569190 genotypes; a. overall association, b. association with childhood onset (<20 years), c. association with adult onset (≥20 Years). * P<0.05.

Two alleles (C and T) and three respective genotypes (CC, CT and TT) were readily detected for the CD14 promoter region at location -159 relative to transcription start site (Figure-3A&B). Genotype frequencies in cases and controls did not show any significant departure from the Hardy-Weinberg equilibrium (p = 0.89 and 0.92, respectively). The frequency of heterozygous (CT) genotype was highest than either of homozygotes (CC and TT)in both case and control populations (Table-3a). There is no significant difference in the genotype or allelic frequencies for the CD14 polymorphism between asthmatic patients and controls (p= 0.178 and 0.177 respectively). The patients included in this analysis were all skin test positive and among them, there was a significant difference of allele and genotype distribution for CD14 polymorphism between childhood onset (<20 years) and adult onset groups (≥ 20 years) (p=0.004 and 0.001 respectively) (Table-3b) and across individuals with increase of disease severity (p=0.002 and 0.009 respectively) (Table-3c). The homozygous CC genotype and also C allele at CD14 -159 were predominant in patients with childhood onset of asthma compared to those of adult onset and in patients with moderate and severe form of the disease than those with a mild form of asthma.

### Association of CD14 promoter region genotypes with IgE levels

Total serum IgE level of case subjects were also tested for associations with SNP genotypes of the CD14 promoter region (Figure-3C). There was a significant increase (P=0.007) in total serum IgE levels in carriers of CC genotype compared to the CT and TT. However, when the patients were sub-divided according to age of disease onset, the differences in serum IgE levels remained significant (P=0.002) in the early onset group only, but in the late onset group of our study population, this association did not reach to statistical significance (p=0.868).

## Discussion

With a background of increased incidence of bronchial asthma in Kolkata population, this study aimed to analyze environmental and genetic factors influencing such multifactorial disease condition. Among total 11 common out-door aero-allergens sensitizers (6 pollen and 5 molds), the most predominant one found in this analysis was *Cocos nucifera* followed by *Caesalpenia sp, Caricapa paya* etc, all of which belong to pollen category. This finding is in agreement with our previous study^2^ indicating that pollen category is a more predominant aero-allergen over mold category in out-door air of Kolkata. On the contrary, mold category was found to be more common over pollen among asthmatic patients of South-Western Iran^18^ indicating importance of geographic location and associated conditions in this respect. A cross reactivity of *Cocos nucifera* with *Areca catechue, Phoenix sylvestris* and some other members of *Palmacae* family was reported^19^ from Eastern India. Surprisingly, very low skin sensitivity of both *Areca* and *Phoenix* as compared to *Cocos nucifera* did not support such cross reactivity in our study population. In the present study, significantly higher numbers of skin test positive patients along with concordant higher level of total serum IgE were found among child and adolescents than in adults and this was preliminarily, in accordance, to the idea^20^ of decline of asthma incidence and severity with age (Cut off values for normal IgE leveling blood: 4–10 years, <250IU/ml; 10–16 years, <200IU/ml;adults, <100IU/ml^21^). However, significant difference of skin test sensitivity and total IgE between childhood onset and adult onset groups imposed more importance on age of disease onset and some differential genetic interplay might be involved forsuch phenotypic heterogeneity. Similarly, Segala et al (2000)^22^ found phenotypic heterogeneity between childhood onset and adult onset asthma and reported comparatively more severity in adults having their disease onset in childhood.

Furthermore Craig et al (2010)^23^ also reported higher frequency of negative skin test and lower level of total serum IgE in patients with late onset asthma. Among the reported genetic polymorphisms involved in allergic manifestations through modulating TH2 mediated IgE response, CD14 C-159T was most widely studied. We found no significant difference in the allele and genotype frequencies of CD14 promoter polymorphism between asthmatic subject and healthy control; therefore, the gene did not seem to constitute a risk factor for the disease development. Similar findings have also been reported by Tan et al (2006)^24^ and Buckova et al (2001)^4^ in different IgE mediated allergic manifestations. Association of CD14 with asthma severity was widely explored in different population with conflicting results: Baldini et al (1999)^9^ reported that C allele of the CD14 polymorphism was associated with atopy in non-hispanic white children as CC genotype was significantly more frequent among skin test positive individuals and associated with higher serum IgE than CT and TT. Similar finding was reported by Buckova et al (2003)^4^ in Czech children sensitized to mold allergen. On the contrary Perin et al (2011)^25^ reported association of C allele of CD14 C(-159)T with severity of non-atopic asthma in Slovenian children and Kedda et al (2005)^12^ found no association of the polymorphism with asthma or asthma severity in an Australian adult population.

In present study with 220 skin test positive asthma patients, C allele was significantly more frequent in childhood onset group only and CC genotype was significantly correlated with higher level of total serum IgE, whereas no such significant association was found in adult onset group. Therefore, childhood onset and adult onset of asthma might be considered as specific sub-sets of the disease in which differential genetic predisposition might be interplaying and accounting for their phenotypic heterogeneity. This might also be an explanation to the inconsistent results reported by different authors on association of CD14 in atopic phenotype in addition to ethnicity. However, further study is warranted in this respect to establish the genetic heterogeneity existing between childhood onset and adult onset of asthma.

## Conclusion

Aero-allergens of pollen category seemed to be more prevalent and cause more severe sensitization in Kolkata metropolis than that of mold. This might have implications in designing preventive strategies. Childhood onset asthma seemed to be more severe than that of adult onset and this might have some genetic background. Therefore effective treatment plan should be designed separately for these two disease sub-sets in accordance.
